# Obesity Reduces Bone Density Associated with Activation of PPARγ and Suppression of Wnt/β-Catenin in Rapidly Growing Male Rats

**DOI:** 10.1371/journal.pone.0013704

**Published:** 2010-10-28

**Authors:** Jin-Ran Chen, Oxana P. Lazarenko, Xianli Wu, Yudong Tong, Michael L. Blackburn, Kartik Shankar, Thomas M. Badger, Martin J. J. Ronis

**Affiliations:** 1 Department of Pediatrics, University of Arkansas for Medical Sciences, Little Rock, Arkansas, United States of America; 2 Department of Physiology and Biophysics, University of Arkansas for Medical Sciences, Little Rock, Arkansas, United States of America; 3 Department of Pharmacology and Toxicology, University of Arkansas for Medical Sciences, Little Rock, Arkansas, United States of America; 4 Arkansas Children's Nutrition Center, Little Rock, Arkansas, United States of America; Universidade Federal do Rio de Janeiro, Brazil

## Abstract

**Background:**

It is well established that excessive consumption of a high fat diet (HFD) results in obesity; however, the consequences of obesity on postnatal skeletal development have not been well studied.

**Methodology and Principal Findings:**

Total enteral nutrition (TEN) was used to feed postnatal day 27 male rats intragastrically with a high 45% fat diet (HFD) for four weeks to induce obesity. Fat mass was increased compared to rats fed TEN diets containing 25% fat (medium fat diet, MFD) or a chow diet (low fat diet, LFD) fed ad libitum with matched body weight gains. Serum leptin and total non-esterified fatty acids (NEFA) were elevated in HFD rats, which also had reduced bone mass compared to LFD-fed animals. This was accompanied by decreases in bone formation, but increases in the bone resorption. Bone marrow adiposity and expression of adipogenic genes, PPARγ and aP2 were increased, whereas osteoblastogenic markers osteocalcin and Runx2 were decreased, in bone in HFD rats compared to LFD controls. The diversion of stromal cell differentiation in response to HFD stemmed from down-regulation of the key canonical Wnt signaling molecule β-catenin protein and reciprocal up-regulation of nuclear PPARγ expression in bone. In a set of *in vitro* studies using pluripotent ST2 bone marrow mesenchymal stromal cells treated with serum from rats on the different diets or using the free fatty acid composition of NEFA quantified in rat serum from HFD-fed animals by GC-MS, we were able to recapitulate our *in vivo* findings.

**Conclusions/Significance:**

These observations strongly suggest that increased NEFA in serum from rats made obese by HFD-feeding impaired bone formation due to stimulation of bone marrow adipogenesis. These effects of obesity on bone in early life may result in impaired attainment of peak bone mass and therefore increase the prevalence of osteoporosis later on in life.

## Introduction

Although it is well established that excessive consumption of a high fat diet (HFD) results in obesity, the consequences of obesity on development, maturation and remodeling of the skeletal system have not been well studied [1], [2]. Body weight (total mass relative to height) can be a strong determinant of bone mass, reflecting adaptation of skeletal remodeling to loading. However, if the confounding factor of body weight is adjusted for, a strong but inverse association between percent fat mass and bone mass is observed [3], suggesting that body composition may be just as important a determinant of bone quality as total body mass. Although somewhat controversial, it has been hypothesized that increased body fat has a negative effect on attaining peak bone mass and bone mineral content [4], [5]. While the relationship between obesity and bone loss and the underlying mechanisms involved are still poorly understood, it is clear that obese children have a significantly increased fracture risk [6] and a direct demonstration of the effects of dietary-induced obesity on bone loss is required.

Bone marrow surrounds trabecular elements of the skeleton and is composed of pluripotent stromal cells. Stromal cells are regulated by a number of factors. When osteoblast differentiation signals, such as Runx2 and Wnt/β-catenin are activated, stromal cells are driven into the osteoblast lineage [7]. In contrast, entry of stromal cells into the adipocyte lineage occurs through activation of the nuclear receptor peroxisome proliferator-activated receptor-gamma (PPARγ). Since bone and fat cells share a common origin, a switching mechanism in mesenchymal stromal cells (MSCs) may explain some previous observations in which factors enhance bone marrow adipogenesis at the expense of osteoblast differentiation [8]. It is not known how dietary-induced obesity affects osteoblast or adipocyte differentiation from MSCs. However, we do know that increased adipose tissue mass leads to an increase in release of biologically active factors, such as adipokines and free fatty acids (FFAs), which may affect this process. Although the mechanism by which FFAs influence the development of chronic diseases is not clearly understood, FFAs might be a candidate to produce bone loss [9], because decreased bone formation [10] and increased bone resorption [11] have been shown under conditions of hyperlipidemia. Epidemiological studies also suggest that bone mineral density is significantly related to serum lipid profiles [12]. Bone biopsies from rodents [13] and patients [14] with degenerative bone disorders such as osteoporosis have revealed fat accumulation in marrow and this may be accompanied by decreases in fatty acid (FA) unsaturation [15]. Leptin (the major adipokine produced by fat cells) is another potential link between obesity and bone mass. Leptin has been shown to regulate bone mass indirectly via stimulation of bone resorption through a neuroendocrine circuit [16]. In addition, functional leptin receptors have recently been described in bone marrow stromal cells and suggested to have multiple direct actions, dependent on differentiation status [17].

In the current study, we utilized a total enteral nutrition (TEN) rat model to feed different diets containing differing amounts of fat during early life. We tested the hypothesis that HFD-induced obesity may impair bone development through elevated FFA levels by increasing bone marrow adipogenesis at the expense of osteoblast differentiation. We show that HFD-induced obesity favors activation of PPARγ while suppressing Runx2 and β-catenin in bone and pre-osteoblasts. The changes in differentiation of adipocytes and osteoblasts observed in bone *in vivo* of these obese animals were also demonstrated *in vitro* after exposing mesenchymal stromal cells to serum from HFD-induced obese animals or to an artificial mixture of purified FAs which mirrors the composition of non-esterified free fatty acids (NEFA) appearing in serum from HFD-induced obese rats.

## Results

### Body Weights, Body Fat and Bone Composition

**Figure 1:**
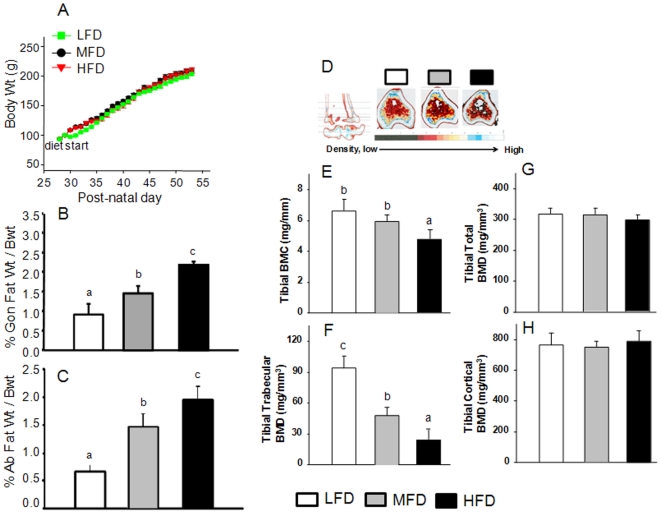
Effect of increasing dietary fat on body weight and composition in male rats. LFD, low fat diet (control pelleted AIN-93G 14% fat diet); MFD, medium fat TEN diet (25% fat diet); HFD, high fat TEN diet (45% fat diet). (A), growth curves from three different diet groups. (B), visceral (gonadal fat) % body weight and (C), abdominal fat % body weight (D), Representative pseudocolored images of tibial peripheral quantitative CT scans (pQCT) (slice 3) from LFD, MFD and HFD rats. Color changes from blue, yellow, red, to gray represent decreases in bone density. Decreased bone mass in male rats fed HFD can be visualized. (E), total tibial bone mineral content (BMC). (F), tibial trabecular bone mineral density (BMD). (G), total tibial BMD. (H), tibial cortical BMD. Data bars are expressed as mean ± SEM (n = 6/group). Means with different letters differ significantly from each other at P<0.05, a<b<c as determined by two-way ANOVA followed by Student-Newman-Keuls post hoc analysis for multiple pairwise comparisons.

TEN was utilized to tightly match body weight gains in all three diet groups. However, after 4 weeks of feeding, gonadal and retroperitoneal adipose tissue (percent body weight) differed and was lowest in the LFD group and highest in the HFD group (P<0.05) (**Figure 1**). Trabecular BMC was lower (P<0.05) in the HFD compared to either the LFD control or MFD groups (**Figure 1E**). Importantly, trabecular BMD was inversely associated with dietary fat content and the observed retroperitoneal and gonadal fat accumulation independent of body weight (**Figure 1F**). There were no differences in total and cortical BMD among groups (**Figure 1G, H**) indicating that short term HFD-induced obesity may initially effect trabecular bone compartment, a site of relatively high bone turnover, before eventually effecting total and cortical BMD.

### Adipogenesis in Bone

**Figure 2:**
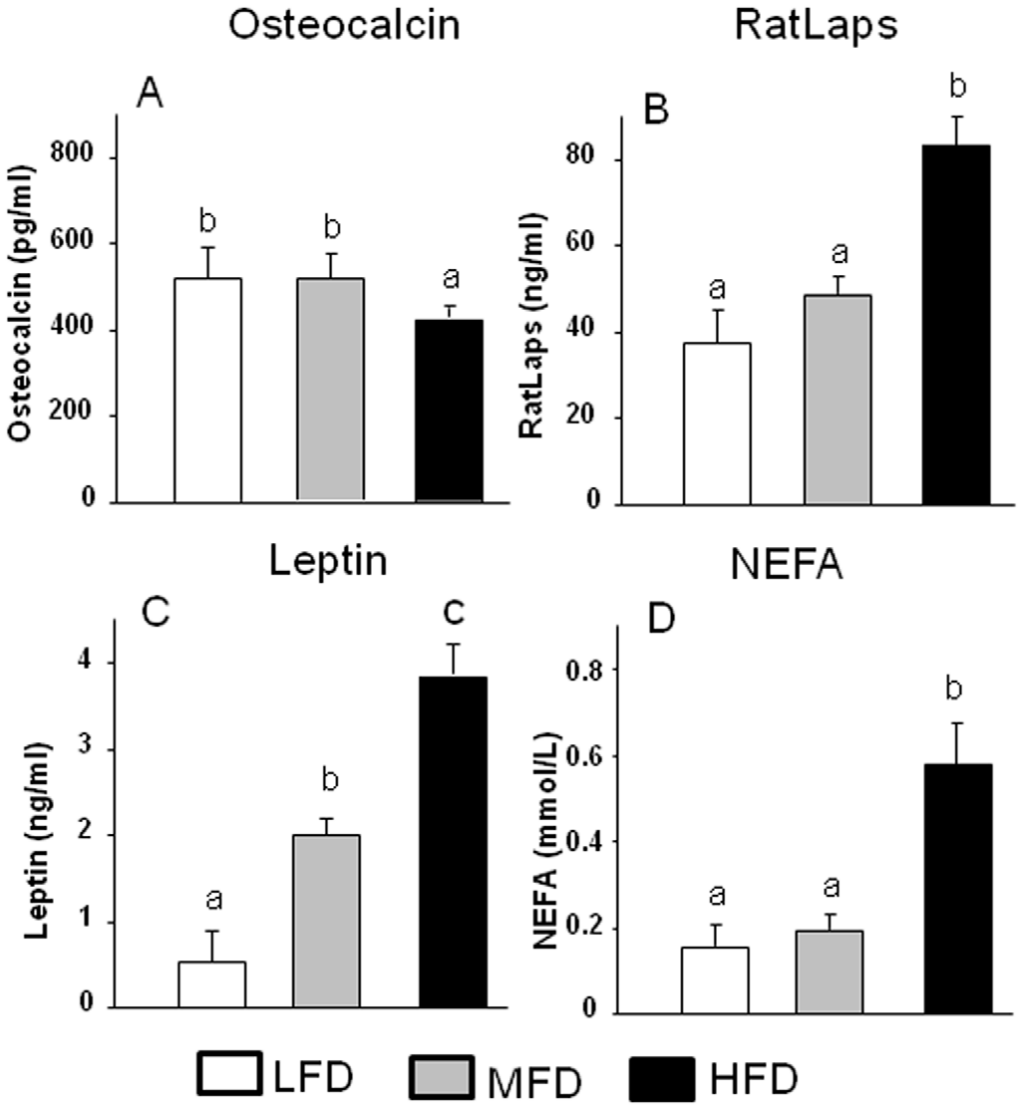
Effects of feeding HFD on serum leptin, non-esterified fatty acids (NEFA) and bone turnover markers. Serum bone formation marker osteocalcin (A), resorption marker RatLaps (B), leptin levels (C), and NEFA levels (D) were measured using standard ELISA methods. Data bars are expressed as mean ± SEM (n = 6/group). LFD, low fat diet (control pelleted AIN-93G 14% fat diet); MFD, medium fat TEN diet (25% fat diet); HFD, high fat TEN diet (45% fat diet). Means with different letters differ significantly from each other at p<0.05, a<b<c as determined by two-way ANOVA followed by Student-Newman-Keuls post hoc analysis for multiple pairwise comparisons.

**Figure 3:**
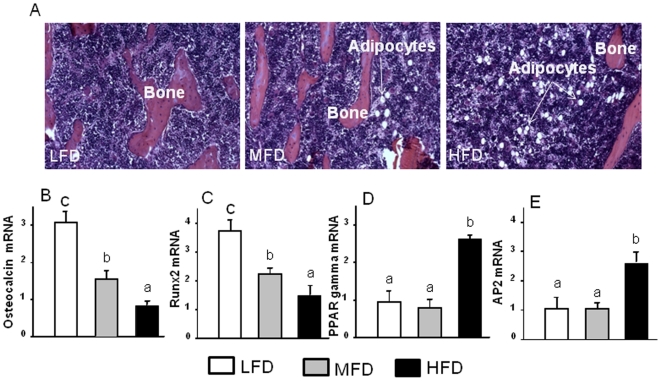
Increased adipogenesis in bone and bone marrow in HFD-induced obese animals. (A), Represented H&E staining picture of increased bone marrow adiposity in tibial bone section from HFD-induced obese animals (10x). (B) and (C), osteocalcin and Runx2 gene expression measured using real-time RT-PCR. (D) and (E), PPARγ and aP2 gene expression measured using real time RT-PCR. Total RNA was isolated from femur of each animal after bone marrow aspiration for RT-PCR. LFD, low fat diet (control pelleted AIN-93G 14% fat diet); MFD, medium fat diet (25% fat diet); HFD, high fat TEN diet (45% fat diet). Data bars are expressed as mean ± SEM (n = 6/group). Means with different letters differ significantly from each other at p<0.05, a<b<c as determined by two-way ANOVA followed by Student-Newman-Keuls post hoc analysis for multiple pairwise comparisons.

To determine if obesity affected balance between osteoblastogenesis and adipogenesis in the bone marrow, we measured bone turnover markers, obesity associated factors in rat serum and expression of key genes in bone tissue. Consistent with previously observed enhanced osteoclastic bone resorption in obese animals [18], we found the serum bone resorption marker RatLaps (C-terminal telopeptide of rat type I collagen) to be greater in the HFD-induced obese group than in the MFD or LFD groups (P<0.05; **Figure 2B**). On the other hand, lower serum levels of the specific bone formation marker osteocalcin was also found in the HFD-induced obese animal group compared to both MFD and LHD groups (P<0.05;**Figure 2A**). Serum leptin levels are positively associated with body fat (**Figure 2C**). However, increases in serum NEFA were only significant in the HFD-induced obese animal group compared to LFD group. NEFA were presumably derived in part from dietary lipid and in part from hydrolysis of triglycerides in adipose tissue (**Figure 2D**). As can be seen from the H&E sections depicted in **Figure 3A**, bone marrow from HFD-induced obese animals had a higher accumulation of fat than the other two groups (P<0.05). Real-time RT-PCR analysis of mRNA levels of adipocyte-specific genes PPARγ and aP2 in bone, following aspiration of bone marrow cells, confirmed the histological findings (**Figure 3D and E**). mRNA levels of bone forming gene osteocalcin and the osteoblast differentiation transcription factor Runx2 were inversely associated with the amount of fat intake (**Figure 3 B and C**; P<0.05).

### PPARγ and β-catenin

**Figure 4:**
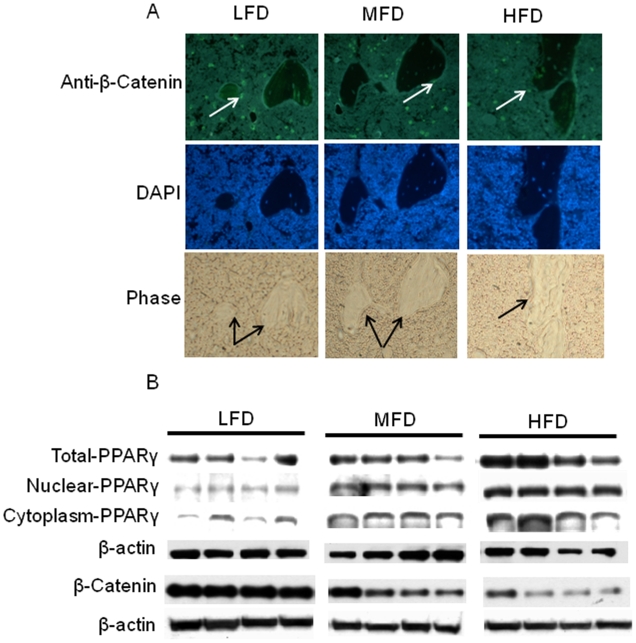
Increased PPARγ but decreased β-catenin protein expression in bone from HFD-induced obese rats. (A) Representative pictures of immunostained tibial bone sections using an anti-mouse β-catenin polyclonal antibody. White arrows indicate immunostained β-catenin, and black arrows indicate bone tissues. (B), Western blots of PPARγ (from total protein lysates, nuclear and cytoplasmic fractions), β-catenin and β-actin are depicted for four samples from LFD, MFD and HFD groups. LFD, low fat diet (control pelleted AIN-93G 14% fat diet); MFD, medium fat TEN diet (25% fat diet); HFD, high fat TEN diet (45% fat diet). Proteins were isolated from long bone after aspiration of bone marrow cells.

In an effort to decipher the molecular signaling involvement in bone leading to the increased in bone marrow adiposity and decreased bone formation in HFD-induced obese animals, we examined the PPARγ and β-catenin protein expression in bone without bone marrow cells and bone marrow. PPARγ and β-catenin are well known adipogenic and osteoblastogenic signaling molecules, respectively. Although we could not identify the specific cell type, immunochemistry of β-catenin from tibial bone sections revealed that expression of β-catenin was lower in the HFD-induced obese animals compared to the LFD control group (P<0.05; **Figure 4A**). Results from western blots of total proteins isolated from femur, with bone marrow cells removed, confirmed that β-catenin protein expression was significantly down-regulated in the HFD-induced obese animals (**Figure 4B**). β-Catenin protein expression was lower in the MFD group compared with LFD controls, but was higher in this group than the HFD-induced obese animals (P<0.05; **Figure 4B**). The expression of PPARγ protein in bone was opposite to β-catenin. The expression of PPARγ protein, especially nuclear fractions from bone from the HFD-induced obese animals, was higher than in either of the other two groups (P<0.05; **Figure 4B**). These data suggest that HFD-induced obesity reciprocally regulates osteoblastogenesis and adipogenesis.

### Serum from HFD-Induced Obese Rats and an Artificial Mixture of FAs Suppress Osteoblast Differentiation *In Vitro*

**Figure 5:**
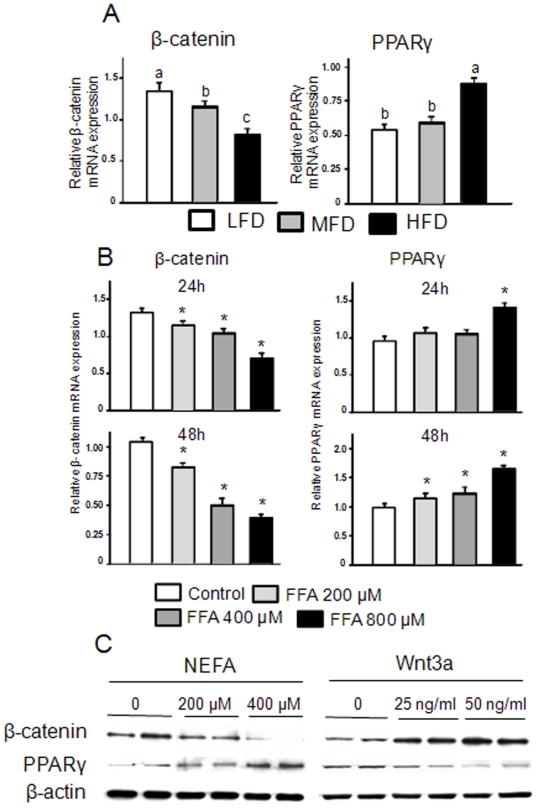
Serum from HFD fed rats and an artificial FA mixture down-regulate β-catenin but up-regulate PPARγ in ST2 cells, Wnt3a does the opposite. (A), ST2 cells were treated with 2% serum from LFD, MFD or HFD rats for 3 days. Cell RNAs were isolated and real-time PCR was performed for β-catenin and PPARγ. (B), ST2 cells were treated with three different concentrations of FAs (200, 400 or 800 µM) for 24 h and 48 h. Cell RNAs were isolated and real-time PCR was performed for β-batenin and PPARγ. (C), ST2 cells were treated with a FA mixture and Wnt3a respectively for 24 h. Cell protein lysates were collected, and western blots were performed for β-catenin, PPARγ and β-actin in duplicates. Data bars are expressed as mean ± SEM (n = 3/treatment). Means with different letters differ significantly from each other at P<0.05, a>b>c, *, P<0.05, *versus* control as determined by two-way ANOVA followed by Student-Newman-Keuls post hoc analysis for multiple pairwise comparisons.

**Figure 6:**
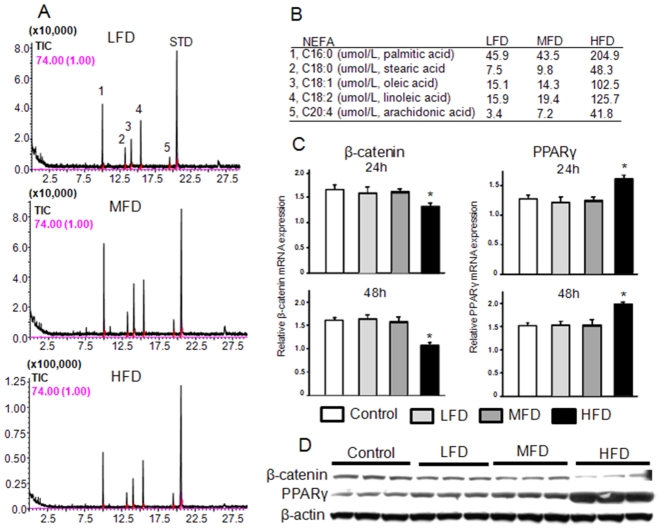
An artificial NEFA mixture based on the ratio and concentrations of FAs appearing in serum from obese rats reciprocally regulates β-catenin and PPARγ expression in ST2 cells. (A), GC-MS analysis of NEFA. To measure NEFA composition, 100 µl of serum from LFD, MFD or HFD rats was characterized and quantified by a Shimazu QP-2010 GC-MS system after TLC separation. (B), Mean concentrations of the 5 major FAs in NEFA are presented. (C), ST2 cells were treated with a NEFA mixture based on individual FA concentrations appearing in serum in LFD, MFD and HFD rats for 24 and 48 h. Real-time RT-PCR of β-catenin and PPARγ mRNA expressions. (D) Western blot analysis of β-catenin and PPARγ (n = 3). LFD, low fat diet (control pelleted AIN-93G 14% fat diet); MFD, medium fat TEN diet (25% fat diet); HFD, high fat TEN diet (45% fat diet). Data bars are expressed as mean ± SEM (n = 3/treatment). *, P<0.05, *versus* control (vehicle treatment) as determined by t-test.

**Figure 7:**
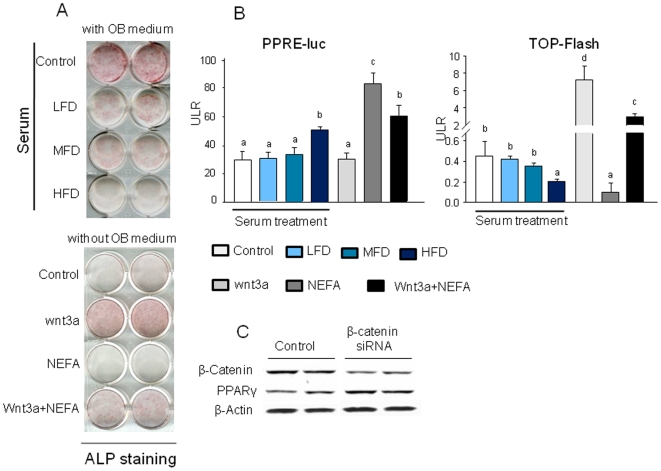
Serum from HFD-induced obese rats and an artificial FA mixture suppress osteoblast differentiation. (A), ST2 cells were cultured in 12 well plates. Cells were treated with 2% serum from LFD, MFD or HFD rats, 50 ng/ml Wnt3a, 400 µM FAs and their combination for 7 days in the presence or absence of osteogenic medium. Alkaline phophatase staining was performed. (B), 2% serum from HFD-induced obese rats and an artificial FA mixture significantly decreased TCF/LEF-dependent transcription of a luciferase reporter gene (TOPFLASH) in C2C12 osteoblastic cells compared with cells treated with LFD serum. Luciferase activity in C2C12 cells transfected with a PPRE-luc reporter construct and treated with 2% serum from LFD, MFD or HFD-fed rats, 50 ng/ml Wnt3a, 400 µM FAs and their combination for 24 h. (C), β-catenin gene was knock down using β-catenin siRNA in ST2 cells. After 24 h of β-catenin siRNA, cell proteins were collected and western blot was performed for β-catenin and PPARγ. Bars are expressed as mean ± SEM in triplicates. *, P<0.05, *versus* control by ANOVA followed by Student-Newman-Keuls post hoc analysis for multiple pairwise comparisons.

**Figure 8:**
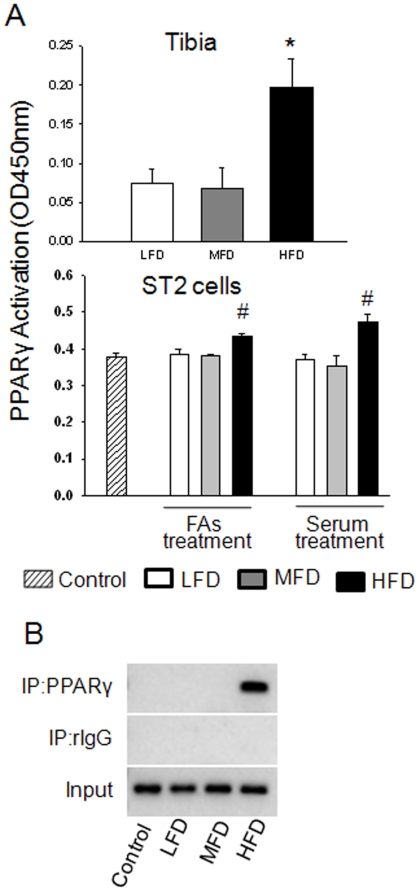
Activation of PPARγ and regulation of its target gene. (A), nuclear proteins were extracted from rat tibia and from ST2 cells treated with rat serum and FAs mixture using 10 cm dish. TransAM was performed for activity of PPARγ. (B) ST2 cells were treated for 24 h with FAs mixture according to the concentrations that appeared in animal circulation. ChIP of mouse aP2 enhancer elements by specific anti PPARγ antibody. Control, normal cell culture medium; LFD, low fat diet (control pelleted AIN-93G 14% fat diet); MFD, medium fat TEN diet (25% fat diet); HFD, high fat TEN diet (45% fat diet). Bars are expressed as mean ± SEM in triplicates. *, P<0.05, *versus* LFD by ANOVA followed by Student-Newman-Keuls post hoc analysis for multiple pairwise comparisons. #, P<0.05, *versus* control by t-test.

We next examined the ability of serum from the HFD-induced obese rats and of a mixture of FAs to affect osteoblast differentiation. To do this, bone marrow derived mesenchymal stromal ST2 cells were first treated with media containing 2% serum from animals from each diet group for 72 h. Similar to the *in vivo* data, real-time PCR revealed that β-catenin gene expression was down-regulated by serum from the HFD-induced obese animals compared to serum from LFD controls (P<0.05; **Figure 5A**). On the other hand, PPARγ gene expression was up-regulated by HFD-induced obese rat serum (P<0.05; **Figure 5A**). Similar to previous studies with macrophages [19], we examined the effects of FAs (a 2∶1 mixture of palmitate and oleate acids) which are known to be elevated in serum of obese individuals on ST2 cells and found a similar pattern of β-catenin and PPARγ gene expression. After 48 h treatment, β-catenin mRNA was down-regulated in a concentration-dependent manner; whereas, PPARγ was up-regulated by FFAs (P<0.05; **Figure 5B**). Consistent with real-time data, western blot showed an inverse association between β-catenin and PPARγ. When ST2 cells were treated with FAs, down-regulated β-catenin was also accompanied with up-regulated PPARγ (P<0.05; **Figure 5C**). On the other hand, when cells were treated with soluble Wnt3a, a well known β-catenin agonist, up-regulated β-catenin was accompanied with down-regulation of PPARγ expression (P<0.05; **Figure 5C**). To further examine composition and concentration of FAs in serum NEFA from our experimental animals, we used GC-MS after TLC separation (**Figure 6A**). We found that there were 5 major FAs: palmitic, stearic, oleic, linoleic and arachidonic acid in the ratio of 5∶1∶2∶3∶1 in rat serum and roughly 5-fold higher concentrations in HFD-induced obese rats with palmitate as the most prominent FA compared to either of the other two diet groups (**Figure 6B**). We next tested whether the mixture of five FAs based on the ratio and concentration of NEFA appearing in serum from obese rats would regulate β-catenin and PPARγ in pre-osteoblasts and observed up-regulated PPARγ and concomitantly down-regulate β-catenin expression (**Figure 6C, D**). The up-regulation of PPARγ was also found in osteogenic cells, such as calvarial cells isolated from neonatal rat calvaria (data not shown here). ST2 cells were also treated with serum from rats from each diet group or FAs in the presence of either osteogenic (OB) medium or Wnt3a for 8 d. Both serum from the HFD-induced obese rats and FAs suppressed bone specific alkaline phosphatase (ALP) activity as assessed by ALP staining (P<0.05; **Figure 7A**), indicating that osteoblast differentiation was suppressed. We next explored potential mechanisms by which HFD-induced rat serum and FAs could attenuate pro-osteogenic canonical Wnt signaling. While both serum from HFD-fed rats and FAs significantly increased PPARγ promoter activity as determined by a PPRE-luciferase reporter assay in C2C12 cells (**Figure 7B**), β-catenin/TCF-mediated transcription was suppressed (P<0.05; **Figure 7B**). We finally examined whether there is an inter-relation between suppressed β-catenin and activated PPARγ in pre-osteoblasts. C2C12 cells were transfected with siRNA against β-catenin. Silencing of β-catenin in pre-osteoblasts increased expression of endogenous PPARγ (**Figure 7C**), indicating the possible existence of switch programs in pre-osteoblasts that direct differentiation to either osteoblasts or adipocytes under appropriate stimuli. Finally, we have examined whether the increased expression of PPARγ leads to increased activity and transcriptional regulation of target genes. Sensitive TransAM^TM^ transcription factor ELISA was performed and DNA binding for activated PPARγ transcription factor was analyzed using samples from both *in vivo* and *in vitro*. As data depicted in Figure 8A, transcriptional factor abundance was significantly increased in bone from obese animals and in pre-osteoblasts treated with FAs and serum from obese animals. To further detect whether FAs enhances binding of PPARγ and its target genes, ChIP assay was carried out on the mouse aP2 gene (**Figure 8B**). We used an antibody against mouse PPARγ and subsequent PCR amplification of adjacent PPRE in the enhancer of the murine aP2 (a known target gene for PPARγ) gene. We found that there was a pronounced increase in the binding of PPARγ to the aP2 enhancer in ST2 cells treated with FAs. These data indicated that HFD-induced obesity and FAs not only increase PPARγ expression but also its transcriptional activity.

## Discussion

Bone development during the post-natal period is largely influenced by diet intake, intrinsic hormonal milieu and physical stimulus, although genetics or pre-natal programming may also play an important role. Positive correlations have been observed between body mass (weight or body mass index) and bone mineral density, particularly at weight-bearing sites [20]. Several recently published studies have highlighted a negative association between obesity and either bone density or quality [21], [22], however, none of these previous studies demonstrated a direct effect of HFD-induced obesity on bone growth. It needs to be noted that body composition varies widely among some populations with the same body mass [23]. We used TEN to carefully feed diets that differed in composition and because we could regulate the amount of diet fed, we could infuse varied levels of total calories to establish groups of rats which had of the same body weight, but differed significantly in body composition to mirror this human situation. The TEN model provides all the nutrients for rats recommended by the National Research Council, it allows precise control of caloric intake and manipulation of percentage of dietary fat. We believe that using this model results in several distinct advantages over other recent studies of obesity and bone. It not only presents a highly reproducible and precise model to control calorie and fat intake, but also eliminates the additional variable of differences in weight gain. Rats fed the high fat diet (HFD) became obese (higher percent body fat), while rats fed the low fat diet (LFD) gained the same weight, but remained leaner. Using this animal model, we were able to study the balance between adipocyte and osteoblast differentiation from MSCs in bone and bone marrow in response to obesity, independent of body weight. Our findings are consistent with other situations such as aging, osteoporosis and chronic alcohol abuse in which increased bone marrow adiposity accompanies decreased bone formation and bone loss [13], [22]. Both *in vivo* and *in vitro* studies have indicated that increased NEFA associated with HFD feeding-induced obesity may be directly responsible for both increased marrow adipogenesis and decreased osteoblast differentiation.

During the preparation of this manuscript, Bielohuby et al reported that feeding a low-carbohydrate/high fat diet *ad libitum* could result in significantly more visceral and bone marrow fat, induce low BMD, and reduce bone formation in rats [24], which are consist with our current findings. Although these investigators showed similar changes in BMD and bone formation and significant accumulation of fat mass, the dietary fat content was excessive. Fat was 66% and 95% of total calories which is well above the 30-45% commonly consumed by U.S. populations [25] and used in the current study.

Another feature of the current study was the use of serum from treated rats to study cells in culture. The idea is to determine if blood born factors could be identified as bioactive components that mediate the effects on bone observed *in vivo*. We have successfully used serum from animals fed different dietary components, such as soy protein isolate and blueberries, to treat stromal cells *in vitro* in previous bone formation and resorption studies [26], [27]. In the present report, serum from LFD control, MFD and HFD-fed rats was used to treat ST2 or C2C12 cells, and cell differentiation into osteoblasts was examined. While both C2C12 and ST2 cells are derived from bone marrow, compared to committed bipotential C2C12 cells, ST2 cells are pluripotent mesenchymal stem/stromal cells. There were some technical reasons for choosing these two different cells in the study, for example transfection efficiency, however, by using these two cell types, our results implicate the possibility that obesity has effects on both osteoblast differentiation and commitment. Using these cell lines treated with HFD rat serum, we were able to confirm our hypothesis that there is an inverse relationship between differentiation of adipocytes and osteoblasts within the marrow cavity [28], and that this balance is disrupted in obesity.

Since serum from HFD rats drives adipocyte differentiation while at the same time diminishing osteoblast differentiation, we next sought to determine which serum component may be the causal factor. Although leptin may play some role in the effects of high fat feeding *in vivo* [16], its main action on bone turnover is an indirect stimulation of bone resorption via effects on RANKL expression [16]. Direct actions of leptin on bone formation are far less clear and appear to be related to regulation of mineralization [17]. However, evidence has been shown that there are biologically relevant PPARγ ligands produced during adipogenesis [29]. It remains to be determined if these endogenous PPARγ ligands also inhibit osteogenesis. We demonstrated that the concentration and ratio of FAs appearing in serum from HFD-induced obese rats can inhibit osteoblast differentiation. Our data indicate that NEFA appears to reciprocally regulate PPARγ and Wnt/β-catenin expression in stromal cells at the transition level, providing a mechanistic explanation for the association between HFD-induced obesity with increased marrow adipogenesis and increased bone loss. Although we have identified and characterized individual FAs in NEFA from serum of HFD rats, whether the effects of NEFA on osteoblast and adipocyte differentiation are due to particular single FA or synergistic effect of multiple FAs still needs to be further investigated. It is still unclear whether NEFA-induced early stage adipocyte commitment is an event independent of osteoblast differentiation since high levels of functional PPARγ may down-regulate the amount and transcriptional activity of β-catenin. Silencing of endogenous β-catenin in pre-osteoblasts leads to over-expression of PPARγ, indicating that β-catenin acts as a suppressor of PPARγ in the osteoblast differentiation. However, the exact molecular mechanism responsible for the down-regulation of β-catenin by PPARγ induced by NEFA remains to be elucidated. It is possible that there is a direct protein-protein interaction between PPARγ and β-catenin, or there is an additional protein such as TAZ, a transcriptional coactivator with PDZ-binding motif [30] that plays a role in mediating β-catenin down-regulation.

In summary, we have presented results demonstrating impaired bone quality in HFD-induced obesity. This appears to be due at least in part to serum NEFA which can directly affect stromal cell differentiation potential in bone marrow. HFD-induced obesity may gradually impair the well maintained balance between adipocyte and osteoblast differentiation in bone marrow, causing a shift that favors adipogenesis while suppressing osteogenesis. We believe that higher levels of NEFA in serum not only activate PPARγ to stimulate adipogenesis, but also suppress Wnt/β-catenin to inhibit osteogenesis. Our data reveal a novel link between HFD-induced obesity and low bone mass with profound implications for further clinical and mechanistic research. Furthermore, these studies clearly provide evidence to support the hypothesis that body composition (independent of body weight) may be just as important a determinant of bone quality as total body mass.

## Materials and Methods

### Animals and Diets

Time-impregnated female Sprague-Dawley rats (n = 6) (Harlan Industries, Indianapolis, IN) arrived on gestational day 4 and were individually housed in an Association for Assessment and Accreditation of Laboratory Animal Care-approved animal facility at the Arkansas Children's Hospital Research Institute with constant humidity and lights on from 06:00–18:00 hrs at 22°C. All animal procedures were approved by the Institutional Animal Care and Use Committee at University of Arkansas for Medical Sciences (UAMS, Little Rock, AR). The approval ID for this study is 2567. Pregnant rats were fed AIN-93G standard diets made with casein as the sole protein source. Litters from these dams were culled to 5 male and 5 female pups. Male pups at post-natal day 20 were randomly assigned (8 per group) to AIN-93G diets with or without surgery. Experimental diets were started 7 days later following acclimation to surgery. The control, low fat diet (LFD) group (n = 8) was fed an AIN-93G diet (14% fat) *ad libitum* [31]. The remaining rats were surgically implanted with an intragastric cannula and fed by TEN as described previously [32]–[34]. Liquid diets were formulated to contain the nutrients recommended by the National Research Council. Rats were randomly assigned to either high fat (HFD; n = 8) or medium fat TEN diets (MFD; n = 8). The HFD diet contained 25% protein, 45% fat (corn oil), and 30% carbohydrate and the MFD diet contained 25% protein, 25% fat (corn oil), and 50% carbohydrate. Body weights were monitored daily and the infusion rate of TEN diets adjusted daily in order to match the weight gain of TEN rats to that of the *ad libitum*-fed LFD control group. The average amount of diet consumed by TEN animals was 352 Kcal per kilogram of body weight, and by control animals was 346 Kcal per kilogram of body weight. Rats were infused TEN diets from 6 PM to 8 AM (lights off 7 PM to 7 AM) for 4 weeks. At the completion of the experiment, rats were anesthetized by injection with 100 mg Nembutal/kg body weight (Avent Laboratories), followed by decapitation, and serum, left and right tibia, femur, and gonadal and abdominal fat were collected.

### Bone Analysis

Peripheral quantitative computerized tomography (pQCT) was performed on formalin fixed left tibia for bone mineral density measurement using a method well established previously in our lab [32] using a STRATEC XCT 960 M unit (XCT Research SA, Norland Medical Systems, Fort Atkins, WI) specifically configured for small bone specimens. Software version 5.4 was used with thresholds of 570 mg/cm^3^ to distinguish cortical bone and 214 mg/cm^3^ to distinguish trabecular from cortical and sub-cortical bone. Tibial bone mineral density (BMD) and bone mineral content (BMC) were automatically calculated and color images generated. The CVs for these measurements were <2%. The position for pQCT scanning was defined at a distance from the proximal tibia growth palate corresponding to ∼7% of the total length of the tibia. Distance between each scanning was 1 mm, total of 5 scans (5 slices) were carried out. Data were expressed as the mean of three contiguous slices with the greatest trabecular bone density.

### Serum Bone Remodeling Markers, Leptin and NEFA

The serum bone formation marker osteocalcin and the serum bone resorption marker procollagen cross-links RatLaps were measured by Rat-MID™ osteocalcin ELISA and RatLapsTM ELISA, respectively, from Nordic Biosciences Diagnostic (Herlev, Denmark). Serum levels of Leptin (ALPCO Diagnostics, 26 Keewaydin Drive Unit G. Salem, NH) and total non-esterified fatty acids (NEFA) (Wako Diagnostics, Wako Chemicals USA, Inc. VA, USA) were measured using standard ELISA method according to procedures provided from the manufacturer. NEFA composition in serum from HFD and LFD-fed rats was characterized and quantified using a Shimazu QP-2010 GC-MS after TLC separation.

### Bone Histology and Immunofluorescence

H&E staining and immunostaining for β-catenin on decalcified tibia sections were carried out using standard protocol from VectaStain ABC kit (Vector Laboratory, Burlingame, CA, USA). For β-catenin immunostaining, sections were deparaffinized, blocked with normal goat serum in 2% BSA-PBS for 30 min, and incubated with polyclonal antibody to β-catenin for 60 min. After three washes with PBS, sections were incubated with biotinylated secondary antibody, which was labeled with streptavidin-conjugated Alexa 488 (Molecular Probes, Carisbad, CA, USA) and counterstained with DAPI (Molecular Probes, Carisbad, CA, USA) [35].

### RNA Isolation and Real-Time PCR Array

Rat femur bone was harvested followed by removal of marrow cells by aspiration according to methods previously described [36]. RNA from femur tissue were extracted using TRI Reagent (MRC Inc., Cincinnati, OH) according to the manufacturer's recommendation followed by DNase digestion and column cleanup using QIAGEN mini columns [36]. Reverse transcription was carried out using an iScript cDNA synthesis kit from Bio-Rad (Hercules, CA). RNA isolation from *in vitro* cell culture was also described previously [35]. All primers for real-time PCR analysis used in this report were designed using Primer Express software 2.0.0 (Applied Biosystems), and are listed in Table 1.

**Table 1:** Real-Time Reverse-Transcription Polymerase Chain Reaction (RT-PCR) Primer Sequences.

Gene	Forward Primer	Reverse Primer
Rat		
Osteocalcin	AAGCCCAGCGACTCTGAGTCT	GCTCCAAGTCCATTGTTGAGGTA
RUNX2	CCGTGGCCTTCAAGGTTGTA	ATTTCGTAGCTCGGCAGAGTAGTT
PPARγ	CCAAGTGACTCTGCTCAAGTATGG	GTCATGAATCCTTGTCCCTCTGA
aP2	CCAAGCCCAACTTGATCATCAG	TGGTCGACTTTCCATCCCACT
GAPDH	TGAGGTGACCGCATCTTCTTG	TGGTAACCAGGCGTCCGATA
Mouse		
PPARγ	GCTTCCACTATGGAGTTCATGCT	CCGGCAGTTAAGATCACACCTAT
β-Catenin	GATATTGACGGGCAGTATGCAA	AACTGCGTGGATGGGATCTG
GAPDH	GTATGACTCCACTCACGGCAAA	GGTCTCGCTCCTGGAAGATG

### Cell Culture

Bone marrow stromal cell line ST2 cells or C2C12 cells were cultured in α-MEM supplemented with 10% fetal bovine serum (FBS) (Hyclone, Logan, UT), penicillin (100 Units/ml), streptomycin (100 µg/ml), and glutamine (4 mM). ST2 cells in 24- and 6-well plates were treated in the presence or absence of 50 ng/ml of soluble Wnt 3a (R & D Systems, Inc.) in combination with or without 400 µM fatty acids (FAs, oleate and palmitate 1∶2 mixture, Sigma-Aldrich) for 7 days followed by ALP staining and collection of proteins for western blotting according to previously published methods [36]. FAs were dissolved in 95% ethanol at 60°C and then mixed with pre-warmed BSA (10%) to yield a stock concentration of 8 mM. Cells were also treated with a second mixture of FAs, the composition, ratio and concentration of which was based on the composition of NEFA appearing in serum from HFD-fed rats. MEM (Invitrogen, Carlsbad, CA) supplemented with 10% FBS, 1 mM ascorbyl-2-phosphate (Sigma-Aldrich), and 4 mM L-glutamine was used as osteoblast differentiation (OB) medium while MEM supplemented with 10% FBS was used as without OB medium. ST2 cells were also treated with cell culture medium containing 2% serum from HFD-fed rats. Half of the cell culture medium was changed every 2 d.

### Constructs for Luciferase Assay and siRNA

Luciferase reporter constructs were introduced into C2C12 cells by transient transfection using Lipofectamine 2000 (Invitrogen, Carlsbad, CA, USA). PPRE-luciferase (PPRE-luc) expression plasmids were kindly provided by B. Spiegelman, Harvard Medical School, Boston, MA (Addgene plasmid 1015). TCF/LEF-Firefly luciferase reporter plasmid (TOPFLASH) and control reporter containing mutant TCF binding sites (FOPFLASH) were purchased from Upstate Biotechnology. C2C12 cells were plated in 48 well plates and transfected 24 h later with a total of 0.4 µg of DNA. Luciferase activity assays were performed as previously described [34]. β-catenin siRNA was purchased from Santa Cruz Biotechnology, Inc. (sc-44253) and transfection of β-catenin siRNA into C2C12 cells was performed following instruction provided by manufacturer.

### Western Blotting, TransAM™ and ChIP Assay

Right tibia bone tissue proteins and *in vitro* cellular proteins were extracted using a cell lysate buffer described previously [35], [36]. Briefly, after cleaning of surrounding connective tissues and aspiration of bone marrow cells, right tibia was smashed to small pieces using surgical pliers. After adding about 400 µl of cell lysate buffer, bone tissues were homogenized using a tissue homogenizer. Nuclear and cytoplasmic protein extractions were performed using NE-PER Nuclear and Cytoplasmic Extraction Reagents kit purchased from PIERCE (cat# 78833, PIERCE Biotechnology). β-catenin, PPARγ and β-actin in bone tissue and *in vitro* osteoblasts were assessed by Western immunoblotting using goat polyclonal antibody recognizing β-catenin (Cell Signaling), rabbit polyclonal antibody recognizing PPARγ (Cell Signaling) and mouse polyclonal antibody recognizing β-actin (Sigma) followed by incubation with either an anti- rabbit or an anti-mouse antibody conjugated with horseradish peroxidase (Santa Cruz). SuperSignal West Pico chemiluminescent substrate (Pierce) was used for developing blots. Quantitation of the intensity of the bands in the autoradiograms was performed using a ChemiDoc XRS imaging system (Bio-Rad). TransAM™ PPARγ transcription factor ELISA kits were purchased from Active Motif (Actove Motif North America, Carlsbad, California, USA. Cat#40196). Nuclear protein fractions from both tissue and cell samples were used for TransAM assay. The assay was performed according to procedures provided from the manufacturer. ChIP assay was performed basically as described in the protocol of the ChIP assay kit from Active Motif (Cat# 53008&53009). FAs treated ST2 cells were cross-linked with formaldehyde in PBS for 10 min, lysed in lysis buffer provided in the kit. 50 µl of chromatin was incubated with anti-PPARγ antibody from Santa Cruz (sc-7196x). Recovered immunoprecipitates were used as template for PCR of aP2 gene using the following mouse specific primers: aP2-F, CCTCCACAATGAGGCAAATC; aP2-R, CTGAAGTCCAGATAGCTC.

### Statistical Analyses

Data were expressed as means ± standard error. One-way or two way analysis of variance (ANOVA) followed by Student-Newman-Keuls post hoc analysis was used to compare the treatment groups. Values were considered statistically significant at p<0.05.
